# Phylodynamics, vectorial competence and genetic diversity of West Nile virus in Africa: implications for global emergence of West Nile

**Published:** 2011-01-10

**Authors:** Gamou Fall, Mawlouth Diallo, Ousmane Faye, Moussa Dia, Anne Dupressoir, Paolo M de Andrade Zanotto, Amadou A Sall

**Affiliations:** 1 Institut Pasteur de Dakar, BP 220 Dakar, Senegal; 2 University of Sao Paulo, SP, Brazil

## 

West Nile virus (WNV) is a flavivirus (Flaviviridae family) and its transmission cycle involves *Culex* spp. mosquitoes and birds as reservoirs host whereas humans and horses are dead-end hosts. Clinical symptoms of WN human infections range from asymptomatic or mild influenza like disease to severe neurological and meningoencephalitis syndromes.

West Nile is a neglected emerging disease with major breakthrough in 1999 with the introduction of WN virus (WNV) in New York City and the subsequent spread to whole northern America over the last decade causing massive human and animals infection leading to some fatal cases. In Eastern Europe, circulation of WNV with recurrent emergences impacting human and animal health since 1996 is similar to the situation in the USA. Strikingly, in Africa WN appears to have a minor effect despite regular isolations from mosquitoes and vertebrates hosts. In addition, WNV exhibited a great diversity with eight different lineages among which only one (lineage 1) is found worldwide and 4 are present in Africa.

In order to understand factors underlying the different patterns of transmission and processes involved in the emergence of WN in the different contexts, genetic diversity, phylodynamics and vectorial competence of WNV have been studied in Africa. Phylogenetic analysis based on partial and complete genome suggests an interconnection of zoonotic amplifications in Africa with emergence in Europe as well as replacement between lineages over time. Vectorial competence of lineages circulating in Africa for a domestic mosquitoes *Culex quinquefasciatus* showed significant differences between strains of various lineages tested for infections, dissemination and transmission rates. Indeed the different strains can be classified as low, intermediary and high infection profile. Analysis of the transmission patterns with sequences of the strains suggest that glysoylation of the envelope protein of WNV, a key player in the virus entry in the cell, may play an important role. The implications of our findings are discussed in the context of global emergence of WN.

